# Nature relatedness, nature-based physical activity, and positive mental health among Chinese university students: a three-wave longitudinal study

**DOI:** 10.3389/fpsyg.2026.1878240

**Published:** 2026-07-03

**Authors:** Shiqi Liu, Yanli Tan, Liuhong Zang

**Affiliations:** School of Physical Education, Xinjiang Normal University, Urumqi, China

**Keywords:** Chinese university students, environmental psychology, human-nature relations, Nature relatedness, nature-based physical activity, positive mental health, psychological flexibility

## Abstract

**Background:**

University students face increasing challenges in maintaining positive mental health, and nature-based approaches have received growing attention as accessible strategies for wellbeing promotion. However, less is known about the behavioral pathway through which nature-related psychological orientations may translate into later positive mental health.

**Objective:**

This three-wave longitudinal study examined whether nature-based physical activity mediated the prospective association between nature relatedness and positive mental health among Chinese university students. Psychological flexibility was further examined as an exploratory boundary condition of the association between nature relatedness and nature-based physical activity.

**Methods:**

A total of 1,191 Chinese university students provided complete and valid matched data across three waves. Nature relatedness, psychological flexibility, and baseline positive mental health were measured at T1; nature-based physical activity was measured at T2; and positive mental health was measured at T3. Ordinary least squares regression and PROCESS Models 4 and 7 with 5,000 bootstrap samples were used, with sex, age, grade, growth environment, green-space accessibility, and baseline positive mental health controlled.

**Results:**

T1 nature relatedness was prospectively associated with greater T2 nature-based physical activity (B = 0.039, *p* < 0.001), and T2 nature-based physical activity was positively associated with T3 positive mental health after controlling for T1 positive mental health (*B* = 0.395, *p* < 0.001). The indirect effect was statistically significant but small (*B* = 0.015, 95% CI [0.006, 0.026]). Psychological flexibility showed a small first-stage moderating effect (*B* = 0.005, *p* = 0.002; ΔR^2^ = 0.008), and the index of moderated mediation was also small (index = 0.002, 95% CI [0.001, 0.004]).

**Conclusion:**

Nature-based physical activity may represent a modest behavioral pathway linking human-nature connection with later positive mental health. The small interaction effect, limited explained variance in the mediator equation, and observational design indicate that psychological flexibility should be interpreted as an exploratory boundary condition rather than a central causal mechanism.

## 1 Introduction

Universities are increasingly expected to support students' capacities for living well within social and ecological constraints (United Nations, 2015; UNESCO, 2021). Within this setting, physical inactivity and mental health difficulties remain major challenges for young adults. The World Health Organization identifies insufficient physical activity as a preventable behavioral risk and calls for settings-based strategies that make active living easier in daily life (World Health Organization, 2022). Current guidelines also recommend regular moderate-to-vigorous activity as a core component of health promotion (World Health Organization, 2020). At the same time, positive mental health has become a central outcome because it captures emotional balance, life satisfaction, meaning, and adaptive functioning beyond the mere absence of distress (Keyes, 2002). The Positive Mental Health Scale provides a concise way to assess this positive dimension of mental health (Lukat et al., 2016), complementing broader work on subjective and eudaimonic wellbeing (Diener et al., 1999; Ryan and Deci, 2001).

A person-environment perspective suggests that positive mental health may be shaped not only by individual traits but also by everyday interactions with natural and built environments. Nature relatedness refers to a dispositional sense of affective, cognitive, and experiential connection with the natural world (Nisbet et al., 2009). The six-item short form was designed to assess this construct efficiently in survey settings (Nisbet and Zelenski, 2013). Although related constructs such as connectedness to nature have been widely used (Mayer and Frantz, 2004), nature relatedness is particularly useful for this study because it captures a relatively enduring orientation toward nature that may guide daily choices. Meta-analytic evidence indicates that connection with nature is positively associated with happiness and eudaimonic wellbeing (Capaldi et al., 2014; Pritchard et al., 2020).

Several theoretical perspectives explain why contact with nature may matter for mental health. Natural environments can support restoration, stress recovery, and adaptive functioning (Hartig et al., 2014). Attention restoration theory proposes that natural settings help restore directed attention through soft fascination and psychological distance from routine demands (Kaplan, 1995), whereas stress-recovery theory suggests that exposure to natural environments may facilitate psychophysiological recovery (Ulrich et al., 1991). Reviews of green exercise and nature exposure further indicate that outdoor natural environments can contribute to physical and mental wellbeing (Bowler et al., 2010; Thompson Coon et al., 2011). Neurocognitive and ecosystem-service perspectives have also linked nature experiences with emotion regulation and mental health processes (Bratman et al., 2015, 2019).

However, these frameworks often imply that exposure to, or connection with, nature benefits mental health without fully specifying the behavioral pathway through which this association unfolds. Nature relatedness may matter most when it is translated into repeated everyday behavior. Population-based evidence shows that spending time in nature is associated with better health and wellbeing (White et al., 2019), and dose-oriented studies suggest that benefits may depend partly on patterns of nature contact (Shanahan et al., 2016). Physical activity offers a modifiable behavioral route through which nature relatedness may become linked to wellbeing. Evidence from physical activity research supports the role of regular movement in physical and psychological health (Warburton et al., 2006), and meta-analytic findings indicate that physical activity is linked to lower depression and anxiety in non-clinical populations (Rebar et al., 2015). Reviews among younger populations and large-scale epidemiological evidence further support the relevance of active behavior for health (Biddle and Asare, 2011; Ekelund et al., 2016).

In the present study, nature-based physical activity refers to moderate-to-vigorous physical activity performed in outdoor or green-space settings, assessed using a brief vital-sign style approach adapted for a nature context (Craig et al., 2003; Coleman et al., 2012). This operational definition focuses on the behavioral combination of movement and outdoor or green-space exposure rather than on detailed ecological quality. It is therefore best understood as a concise behavioral index of nature-based activity opportunity and engagement, not as a comprehensive measure of activity type, intensity distribution, biodiversity, perceived naturalness, or environmental quality.

A psychological orientation toward nature does not automatically become nature-based physical activity. The theory of planned behavior suggests that attitudes and values require translation into behavioral intentions and action (Ajzen, 1991). Health action approaches also emphasize that motivation must be converted into behavior through self-regulatory processes (Schwarzer, 2008). Self-determination theory similarly indicates that personally meaningful behavior is more likely to be enacted when individuals can align action with values and basic psychological needs (Deci and Ryan, 2000). Psychological flexibility offers a plausible, but exploratory, boundary condition for this translation process. It reflects the capacity to stay in contact with present experience, accept internal discomfort, and act in accordance with personally meaningful values (Hayes et al., 1996, 2006). As a broad aspect of adaptive functioning (Kashdan and Rottenberg, 2010), psychological flexibility has been measured through established instruments such as the Acceptance and Action Questionnaire-II (Bond et al., 2011) and the more context-sensitive Psy-Flex (Gloster et al., 2021).

The present work also builds on conditional process research with Chinese student samples while positioning the model within environmental psychology. Recent three-wave longitudinal research has illustrated how a personal resource can be linked to later competence through a mediator while a self-related factor strengthens the first-stage pathway (Su and Chen, 2026). Other PROCESS studies have examined how personality or behavioral resources shape stress-to-mediator pathways among students (Zuo et al., 2024). Longitudinal moderated mediation work has also shown that adult attachment and physical activity can condition the psychological consequences of earlier adversity (Jiang et al., 2022). Recent studies in Chinese adolescent and student samples have used physical activity as a moderator in models involving experiential avoidance, anxiety, sleep, and internet-related outcomes (Wang et al., 2025; Peng et al., 2025c). Additional work using similar conditional process logic supports cautious interpretation of small interaction effects in student health research (Peng et al., 2025d,b). Another study has applied a comparable moderated mediation framework to anxiety and sleep hygiene among college students (Peng et al., 2025a). Research on digital learning has likewise emphasized that self-regulatory resources may shape how students convert psychological tendencies into adaptive or maladaptive outcomes (Zhang and Xu, 2025).

Two gaps motivate the present study. First, although nature relatedness and wellbeing have been linked in prior research, fewer studies have tested whether nature-based physical activity serves as a time-lagged behavioral mediator among Chinese university students. Second, limited evidence has examined whether psychological flexibility may condition the behavioral translation of nature relatedness into nature-based physical activity. To address these gaps, this study used a three-wave design in which T1 nature relatedness and psychological flexibility were measured first, T2 nature-based physical activity was measured ~5 weeks later, and T3 positive mental health was assessed at the final wave. Baseline positive mental health was also measured at T1 and controlled in the main analyses. We tested four hypotheses: H1, T1 nature relatedness would be positively associated with T3 positive mental health after controlling for T1 positive mental health; H2, T2 nature-based physical activity would mediate the association between T1 nature relatedness and T3 positive mental health; H3, T1 psychological flexibility would show an exploratory first-stage moderation of the association between T1 nature relatedness and T2 nature-based physical activity; and H4, the indirect association from T1 nature relatedness to T3 positive mental health through T2 nature-based physical activity would vary by T1 psychological flexibility.

**Figure 1:**
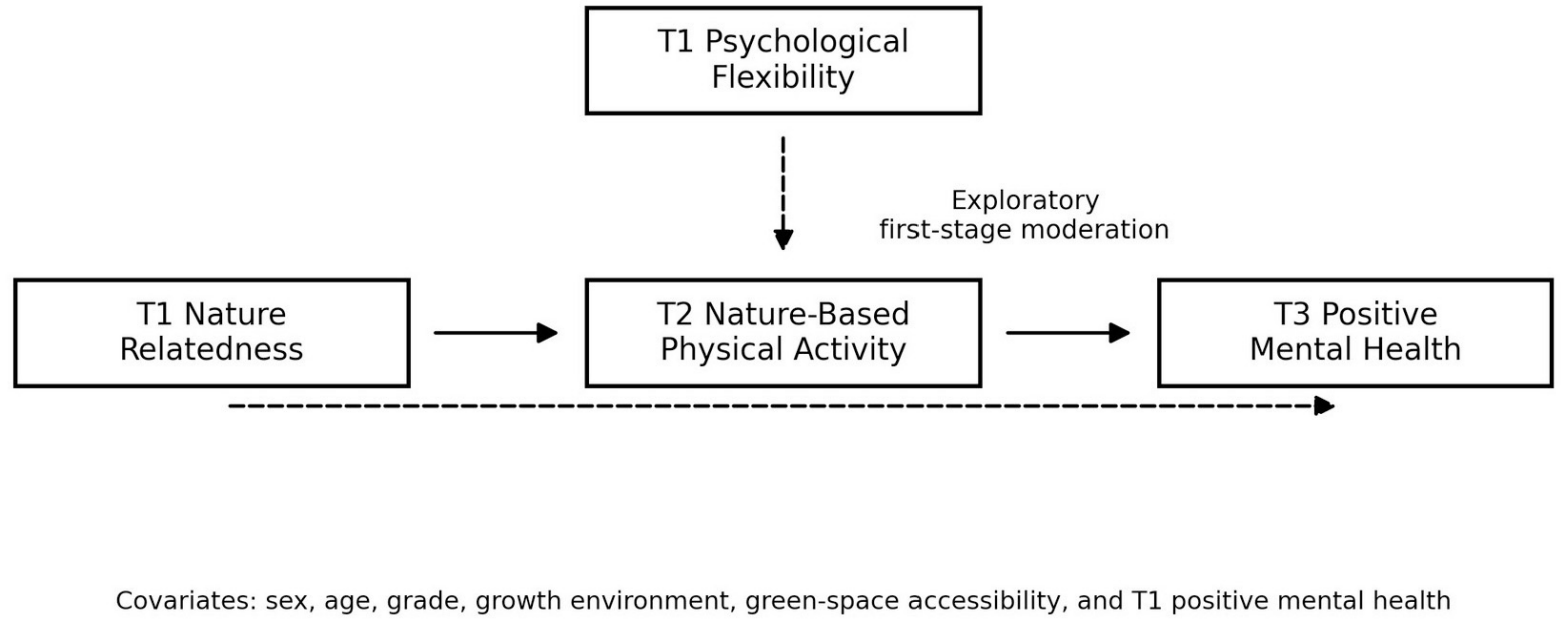
Conceptual model of the proposed longitudinal mediation pathway and exploratory first-stage moderation. Demographic covariates (sex, age, grade, growth environment, and green-space accessibility) and T1 positive mental health were modeled as covariates but are omitted from the figure for readability. PA = physical activity.

The conceptual longitudinal mediation model and exploratory first-stage moderation are presented in Figure 1.

## 2 Materials and methods

### 2.1 Study design and participants

Data were collected during the spring semester of 2026 across three waves, with approximately five-week intervals between adjacent waves. The three waves yielded 1,632, 1,517, and 1,390 returned questionnaires, respectively. At T1, 142 invalid responses were excluded during baseline quality screening, resulting in 1,490 valid baseline responses. Responses were excluded if participants failed the attention-check item, provided an invalid or missing anonymous matching code, had missing data on focal variables, completed the survey in an unrealistically short time, submitted duplicate responses, or showed obvious patterned responding. After quality screening and three-wave matching using anonymous codes, the final analytic sample consisted of 1,191 university students with complete and valid longitudinal data. The lost group in the attrition analysis comprised the 299 valid T1 respondents who were not retained after three-wave matching (1,490–1,191 = 299).

Participants were informed that participation was voluntary and anonymous, and that they could withdraw at any time. The study was conducted in accordance with the Declaration of Helsinki and was approved by the Ethics Committee of Xinjiang Normal University (approval number: XJNU2026LLSC52). Electronic informed consent was obtained before survey completion: participants first read the study information and selected an agreement option before entering the questionnaire; those who did not agree did not continue.

### 2.2 Measures

#### 2.2.1 Nature relatedness

Nature relatedness was measured at T1 using the six-item Nature Relatedness Scale short form. Recent validation work has provided Chinese evidence for the broader Nature Relatedness Scale in university students (Zuo et al., 2025). Because the present study used the six-item short form, the English items were translated, back-translated, and checked by a bilingual research team before data collection. Items were rated on a five-point scale from 1 (strongly disagree) to 5 (strongly agree). Higher total scores indicated stronger psychological connection with nature. In the present sample, Cronbach's alpha was 0.884.

#### 2.2.2 Psychological flexibility

Psychological flexibility was measured at T1 using the six-item Psy-Flex. The Chinese version of the simplified psychological flexibility scale has shown acceptable psychometric properties in Chinese samples (Fang et al., 2024). The present survey wording was prepared through translation, back-translation, and review by bilingual researchers to preserve the meaning of the original items. Items were rated on a five-point scale, with higher total scores reflecting greater psychological flexibility and values-based adaptive responding. Cronbach's alpha in this study was 0.883.

#### 2.2.3 Positive mental health

Positive mental health was measured at T1 and T3 using the nine-item Positive Mental Health Scale. The PMH-scale has been used in a large Chinese student sample in longitudinal research on positive mental health and life satisfaction (Bieda et al., 2019). The Chinese survey wording was prepared through translation, back-translation, and bilingual discussion to ensure conceptual equivalence across waves. Items were rated from 1 (does not apply at all) to 4 (applies very well). Higher total scores indicated better positive mental health. Cronbach's alpha was 0.931 at T1 and 0.925 at T3.

#### 2.2.4 Nature-based physical activity

Nature-based physical activity was measured at T2 using two items. Students reported the number of days in the past seven days on which they engaged in at least 10 min of moderate-to-vigorous physical activity in outdoor or green-space settings and the average duration per active session. Weekly nature-based physical activity minutes were calculated as days multiplied by average minutes per session. The measure was adapted from brief physical-activity screening and vital-sign approaches and was modified to capture activity occurring in outdoor or green-space contexts (Craig et al., 2003; Coleman et al., 2012). In this study, “nature-based” therefore refers to physical activity that combines movement with outdoor or green-space exposure. The measure does not capture activity type, exact intensity distribution, biodiversity, perceived naturalness, or environmental quality, and this limitation is explicitly acknowledged in the Discussion. Because weekly minutes were right-skewed, the main analyses used log-transformed minutes, calculated as ln(minutes + 1).

#### 2.2.5 Covariates

The main models controlled for sex, age, grade, growth environment, perceived green-space accessibility, and baseline positive mental health. A recent stressful event variable measured at T3 was used in sensitivity analysis because it was assessed concurrently with the outcome.

### 2.3 Statistical analysis

Analyses were conducted using ordinary least squares regression and the PROCESS conditional process framework. Reliability was evaluated using Cronbach's alpha. Descriptive statistics included means, standard deviations, medians, ranges, skewness, and kurtosis. Pearson correlations were used to examine bivariate associations among the focal variables. Common method bias was assessed using Harman's single-factor test; this test was interpreted cautiously because it is a limited diagnostic for shared method variance in self-report research (Podsakoff et al., 2003). Unstandardized regression coefficients (B) are reported unless otherwise stated.

A baseline-adjusted longitudinal mediation model was first tested using PROCESS Model 4. T1 nature relatedness was specified as the predictor, T2 nature-based physical activity as the mediator, and T3 positive mental health as the outcome. The first-stage moderated mediation model was then tested using PROCESS Model 7, with T1 psychological flexibility examined as an exploratory moderator of the pathway from T1 nature relatedness to T2 nature-based physical activity. Baseline positive mental health and demographic covariates were controlled. Continuous predictors were mean-centered before the interaction term was created. Indirect effects, conditional indirect effects, and the index of moderated mediation were examined using 5,000 bootstrap samples. Because nature-based physical activity showed high kurtosis, robustness checks for the interaction included HC3 heteroskedasticity-consistent standard errors, 1%/99% winsorization of weekly activity minutes, exclusion of observations with absolute standardized residuals greater than 3 in the mediator equation, and exclusion of influential cases using Cook's distance greater than 4/n. Bootstrap inference for indirect effects followed established mediation recommendations (MacKinnon et al., 2004). Moderated mediation tests followed conditional process guidance (Preacher et al., 2007; Hayes, 2015). Model implementation followed Hayes's regression-based approach (Hayes, 2022).

Because this was an observational longitudinal survey, all mediation and moderation results were interpreted as time-lagged statistical associations rather than evidence of causal effects. Baseline adjustment allowed the analyses to account for earlier positive mental health but did not remove all possible confounding. Longitudinal mediation decisions were guided by recommendations on temporal ordering and bias in mediation models (Cole and Maxwell, 2003; Maxwell and Cole, 2007). Broader longitudinal modeling decisions followed standard longitudinal structural modeling guidance (Little, 2013). Missing-data and attrition issues were interpreted using standard guidance for incomplete data (Enders, 2010; Schafer and Graham, 2002). Additional recommendations for missing data in behavioral research were also considered (Graham, 2009). Interaction interpretation and multivariable regression decisions followed standard regression texts (Aiken and West, 1991; Cohen et al., 2003). Model specification and power considerations were informed by behavioral-science modeling and power-analysis guidance (Kline, 2016; Faul et al., 2009). Reporting transparency was checked against observational-study guidance (von Elm et al., 2007; Vandenbroucke et al., 2007). Multiple-imputation guidance was considered for missing-data transparency, although the primary analysis used complete matched data (Sterne et al., 2009).

## 3 Results

### 3.1 Sample flow, participant characteristics, and attrition analysis

The sample flow and participant characteristics are presented in Table 1.

**Table 1:** Sample flow and participant characteristics.

Item	*n*/M ±SD	%
T1 returned questionnaires	1,632	—
T2 returned questionnaires	1,517	—
T3 returned questionnaires	1,390	—
Final three-wave matched analytic sample	1,191	—
Sex: male	504	42.32
Sex: female	687	57.68
Grade: first-year undergraduate	371	31.15
Grade: second-year undergraduate	290	24.35
Grade: third-year undergraduate	292	24.52
Grade: fourth-year undergraduate	184	15.45
Grade: postgraduate or above	54	4.53
Growth environment: urban	540	45.34
Growth environment: county/town	354	29.72
Growth environment: rural	243	20.40
Growth environment: pastoral/mountain/nature-rich area	54	4.53
Green-space accessibility: very inconvenient	59	4.95
Green-space accessibility: not very convenient	200	16.79
Green-space accessibility: average	390	32.75
Green-space accessibility: relatively convenient	381	31.99
Green-space accessibility: very convenient	161	13.52
Age, M ± SD	20.55 ± 1.94	-

The final analytic sample consisted of students who provided complete and valid data across all three waves after quality screening and anonymous-code matching. For attrition analyses, 142 invalid T1 responses were first excluded from the 1,632 returned questionnaires, yielding 1,490 valid T1 baseline responses; the lost group comprised 299 valid T1 respondents not retained after three-wave matching.

Attrition analyses compared participants retained in the final analytic sample with T1 valid respondents who were not retained after three-wave matching. No significant differences were found in age, sex, growth environment, T1 nature relatedness, T1 psychological flexibility, or T1 positive mental health. Significant differences were observed in grade and perceived green-space accessibility; therefore, these variables were retained as covariates. The attrition analysis is shown in Table 2.

**Table 2:** Attrition analysis comparing retained and lost participants.

Variable	Retained (*n* = 1,191)	Lost (*n* = 299)	Statistic	*p*	Effect size
Age	20.55 ± 1.94	20.73 ± 2.05	*t*_(1, 488)_ = −1.358	0.175	*d* = −0.092
T1 nature relatedness	19.77 ± 4.24	19.67 ± 4.58	*t*_(1, 488)_ = 0.357	0.721	*d* = 0.023
T1 psychological flexibility	18.15 ± 4.27	18.14 ± 4.38	*t*_(1, 488)_ = 0.035	0.972	*d* = 0.002
T1 positive mental health	23.83 ± 5.92	23.32 ± 6.39	*t*_(1, 488)_ = 1.268	0.205	*d* = 0.085
Green-space accessibility	3.32 ± 1.06	3.43 ± 1.11	*t*_(1, 488)_ = −1.520	0.129	*d* = −0.103
Sex	-	-	chi-square(1) = 0.040	0.842	Cramer's V =0.005
Grade	-	-	chi-square(4) = 14.459	0.006	Cramer's V =0.049
Growth environment	-	-	chi-square(3) = 3.192	0.363	Cramer's V =0.027
Green-space accessibility categories	-	-	chi-square(4) = 13.762	0.008	Cramer's V =0.048

Continuous variables were compared using independent-samples *t*-tests and Cohen's *d*; categorical variables were compared using chi-square tests and Cramer's V. Effect sizes were small.

### 3.2 Common method bias, reliability, and descriptive statistics

Harman's single-factor test was conducted using the psychological scale items. The behavioral physical activity index was not included in this factor analysis because it was computed from frequency and duration indicators rather than from interchangeable latent-scale items. The Kaiser-Meyer-Olkin value was 0.967, and Bartlett's test of sphericity was significant, chi-square(435) = 21,066.857, *p* < 0.001. Four factors had eigenvalues greater than 1, and the first unrotated factor explained 37.15% of the variance. These results suggested that severe common method bias was unlikely, although shared method variance could not be completely ruled out.

Reliability coefficients and descriptive statistics are presented in Table 3. Cronbach's alpha values ranged from 0.883 to 0.931, indicating good internal consistency. Nature-based physical activity was treated as a behavioral index and was therefore not evaluated using Cronbach's alpha.

**Table 3:** Reliability and descriptive statistics for study variables.

Variable	Items	Range	M	SD	Median	Min	Max	Skew.	Kurt.	α
T1 NR	6	6–30	19.77	4.24	20.00	8	30	−0.07	−0.29	0.884
T1 PF	6	6–30	18.15	4.27	18.00	6	30	−0.06	−0.22	0.883
T1 PMH	9	9–36	23.83	5.92	24.00	9	36	−0.01	−0.54	0.931
T2 PA days	1	0–7	3.55	1.52	4.00	0	7	−0.25	−0.18	-
T2 PA minutes/session	1	Observed	33.80	19.96	30.00	0	228	1.97	10.43	-
T2 PA weekly minutes	Index	Observed	135.44	115.60	110.00	0	1,368	2.53	14.18	-
T2 PA (ln)	Index	Observed	4.49	1.19	4.71	0.00	7.22	−2.00	5.55	-
T3 PMH	9	9–36	26.06	5.70	26.00	10	36	−0.29	−0.59	0.925

NR, nature relatedness; PF, psychological flexibility; PMH, positive mental health; PA, nature-based physical activity. PA (ln) = ln(weekly minutes + 1). Ranges for PA variables are observed ranges.

### 3.3 Correlations among study variables

The Pearson correlation matrix is shown in Table 4. T1 nature relatedness was positively correlated with T2 nature-based physical activity and T3 positive mental health. T1 positive mental health showed a strong positive association with T3 positive mental health, supporting its inclusion as a baseline covariate.

**Table 4:** Pearson correlations among focal variables.

Variable	1	2	3	4	5
T1 NR	0.884				
T1 PF	0.292^***^	0.883			
T1 PMH	0.321^***^	0.461^***^	0.931		
T2 PA (ln)	0.138^***^	0.006	0.048	-	
T3 PMH	0.313^***^	0.352^***^	0.626^***^	0.121^***^	0.925

NR, nature relatedness; PF, psychological flexibility; PMH, positive mental health; PA, nature-based physical activity. PA was log-transformed.

^***^p < 0.001.

### 3.4 Baseline-adjusted longitudinal mediation

The mediation results are presented in Table 5. T1 nature relatedness was positively associated with T2 nature-based physical activity. T2 nature-based physical activity was positively associated with T3 positive mental health after controlling for T1 positive mental health and demographic covariates. The indirect effect was significant, B = 0.015, 95% CI [0.006, 0.026]. Because the direct effect remained significant, the result indicates a partial indirect association rather than full mediation.

**Table 5:** Baseline-adjusted longitudinal mediation results.

Outcome/ effect	Predictor	B	SE	*t*	*p*	95% CI
M: T2 PA (ln)	T1 NR	0.038	0.008	4.523	<0.001	[0.022, 0.055]
M: T2 PA (ln)	Sex	−0.291	0.069	−4.236	<0.001	[−0.425, −0.156]
M: T2 PA (ln)	Age	−0.018	0.029	−0.625	0.532	[−0.076, 0.039]
M: T2 PA (ln)	Grade	0.046	0.047	0.969	0.333	[−0.047, 0.139]
M: T2 PA (ln)	Growth environment	−0.030	0.038	−0.800	0.424	[−0.104, 0.044]
M: T2 PA (ln)	Green-space accessibility	0.070	0.032	2.179	0.030	[0.007, 0.132]
M: T2 PA (ln)	T1 PMH	0.001	0.006	0.177	0.860	[−0.011, 0.013]
Y: T3 PMH	T1 NR	0.152	0.032	4.763	<0.001	[0.089, 0.214]
Y: T3 PMH	T2 PA (ln)	0.395	0.109	3.620	<0.001	[0.181, 0.609]
Y: T3 PMH	Sex	0.350	0.259	1.349	0.178	[−0.159, 0.858]
Y: T3 PMH	Age	−0.088	0.110	−0.800	0.424	[−0.303, 0.128]
Y: T3 PMH	Grade	0.119	0.177	0.670	0.503	[−0.229, 0.467]
Y: T3 PMH	Growth environment	0.320	0.141	2.269	0.023	[0.043, 0.596]
Y: T3 PMH	Green-space accessibility	−0.020	0.120	−0.164	0.869	[−0.256, 0.216]
Y: T3 PMH	T1 PMH	0.564	0.023	24.961	<0.001	[0.520, 0.608]
Effects	Total effect (c)	0.167	0.032		<0.001	[0.104, 0.229]
Effects	Direct effect (c′)	0.152	0.032		<0.001	[0.089, 0.214]
Effects	Indirect effect (a × b)	0.015	0.005			[0.006, 0.026]

### 3.5 First-stage moderated mediation

The first-stage moderated mediation results are shown in Table 6. The interaction between T1 nature relatedness and T1 psychological flexibility significantly predicted T2 nature-based physical activity, B = 0.005, p =0.002, ΔR^2^ = 0.008. This indicates a statistically significant but small first-stage moderation effect. Therefore, psychological flexibility was interpreted as an exploratory boundary condition rather than a central explanatory mechanism.

**Table 6:** Baseline-adjusted PROCESS Model 7 regression results.

Outcome	Predictor	B	SE	t	p	95% CI
M: T2 PA (ln)	T1 NR	0.039	0.009	4.627	<0.001	[0.023, 0.056]
M: T2 PA (ln)	T1 PF	−0.012	0.009	−1.287	0.198	[−0.029, 0.006]
M: T2 PA (ln)	T1 NR × T1 PF	0.005	0.002	3.085	0.002	[0.002, 0.009]
M: T2 PA (ln)	Sex	−0.286	0.068	−4.191	<0.001	[−0.420, −0.152]
M: T2 PA (ln)	Age	−0.016	0.029	−0.552	0.581	[−0.073, 0.041]
M: T2 PA (ln)	Grade	0.041	0.047	0.873	0.383	[−0.051, 0.134]
M: T2 PA (ln)	Growth environment	−0.027	0.037	−0.713	0.476	[−0.100, 0.047]
M: T2 PA (ln)	Green-space accessibility	0.065	0.032	2.052	0.040	[0.003, 0.128]
M: T2 PA (ln)	T1 PMH	0.004	0.007	0.669	0.504	[−0.008, 0.017]
Y: T3 PMH	T1 NR	0.152	0.032	4.763	<0.001	[0.089, 0.214]
Y: T3 PMH	T2 PA (ln)	0.395	0.109	3.620	<0.001	[0.181, 0.609]
Y: T3 PMH	Sex	0.350	0.259	1.349	0.178	[−0.159, 0.858]
Y: T3 PMH	Age	−0.088	0.110	−0.800	0.424	[−0.303, 0.128]
Y: T3 PMH	Grade	0.119	0.177	0.670	0.503	[−0.229, 0.467]
Y: T3 PMH	Growth environment	0.320	0.141	2.269	0.023	[0.043, 0.596]
Y: T3 PMH	Green-space accessibility	−0.020	0.120	−0.164	0.869	[−0.256, 0.216]
Y: T3 PMH	T1 PMH	0.564	0.023	24.961	<0.001	[0.520, 0.608]

B, unstandardized coefficient. Continuous predictors were mean-centered before forming the interaction term. M-equation *R*^2^ = 0.049; Y-equation *R*^2^ = 0.416; interaction ΔR^2^ = 0.008.

**Figure 2:**
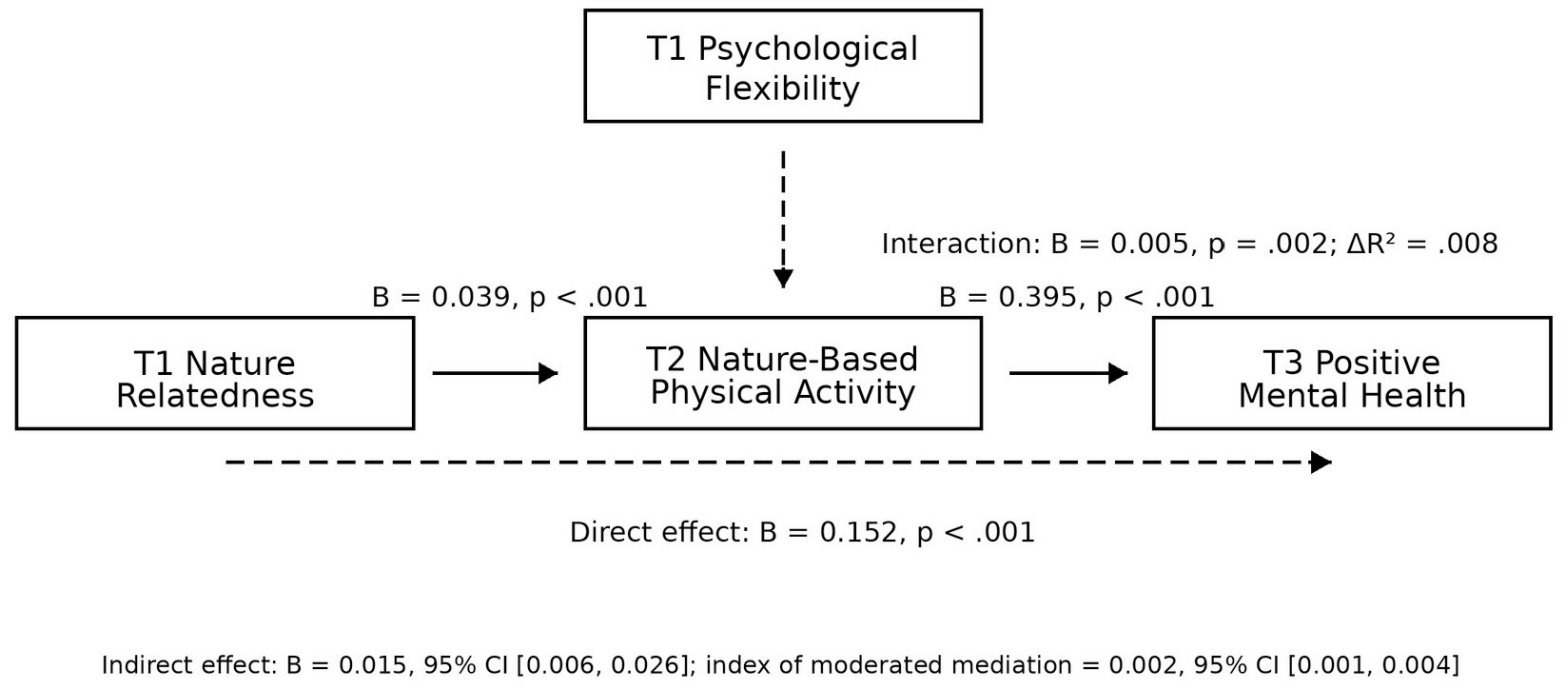
Estimated longitudinal mediation and exploratory first-stage moderated mediation model. Coefficients are unstandardized estimates from the main PROCESS Model 7 analysis. The mediator was log-transformed nature-based physical activity [ln(minutes + 1)]. Demographic covariates and baseline positive mental health were included in the analyses but are omitted from the figure for clarity. PA, physical activity.

Figure 2 summarizes the estimated longitudinal mediation and exploratory first-stage moderated mediation model.

**Figure 3:**
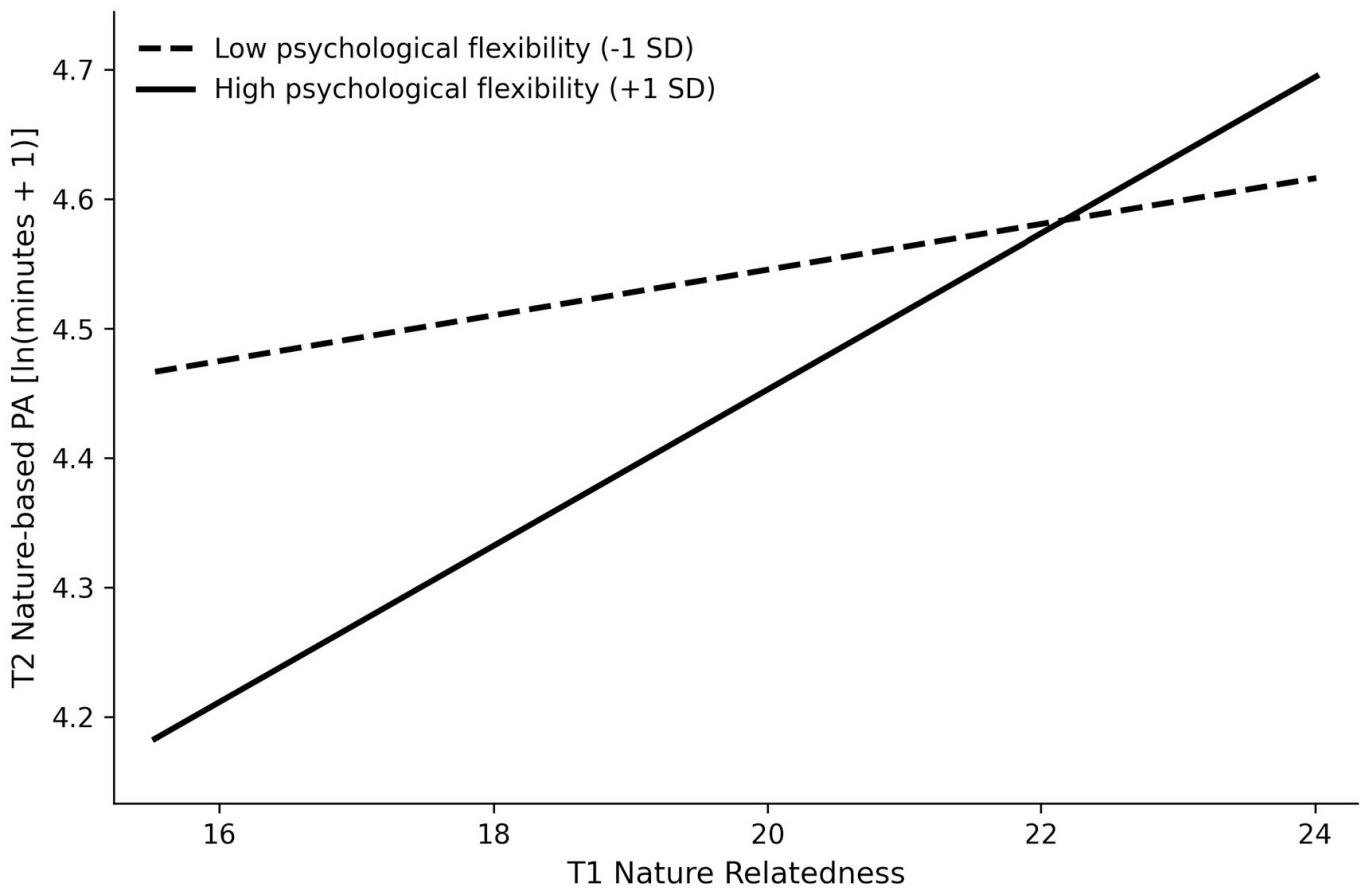
Simple slopes of T1 nature relatedness predicting T2 nature-based physical activity at low and high levels of T1 psychological flexibility. Predicted values were calculated from the moderated regression equation with covariates held at their sample means. Low and high psychological flexibility represent one standard deviation below and above the sample mean, respectively. The x-axis spans T1 nature relatedness from one standard deviation below to one standard deviation above the sample mean; the y-axis shows log-transformed nature-based physical activity [ln(minutes + 1)].

Simple slopes and conditional indirect effects are presented in Table 7 and illustrated in Figure 3. The association between T1 nature relatedness and T2 nature-based physical activity was not significant at low psychological flexibility, but it was significant at mean and high psychological flexibility. The index of moderated mediation was significant, index = 0.002, 95% CI [0.001, 0.004], indicating that the indirect association varied modestly across levels of psychological flexibility.

**Table 7:** Simple slopes and conditional indirect effects.

PF level	Simple slope B	SE	p	95% CI	Indirect effect	Bootstrap 95% CI
Low (−1 SD)	0.016	0.011	0.150	[−0.006, 0.039]	0.007	[−0.001, 0.016]
Mean	0.039	0.009	< 0.001	[0.023, 0.056]	0.016	[0.006, 0.027]
High (+1 SD)	0.062	0.011	< 0.001	[0.040, 0.084]	0.025	[0.011, 0.042]
Index of moderated mediation					0.002	[0.001, 0.004]

PF, psychological flexibility. Low and high levels are defined as one standard deviation below and above the mean. Conditional indirect effects were tested using 5,000 bootstrap samples.

Figure 3 shows the interaction between T1 nature relatedness and T1 psychological flexibility in predicting T2 nature-based physical activity.

### 3.6 Sensitivity analyses

Sensitivity analyses supported the direction of the main findings (Table 8). When raw weekly nature-based physical activity minutes were used as the mediator, the T1 nature relatedness × T1 psychological flexibility interaction remained significant, and raw weekly minutes were positively associated with T3 positive mental health. When guideline-based activity status was used as a binary mediator, the interaction and the association between meeting the 150-min threshold and T3 positive mental health were also significant. In addition, the main findings remained stable after additionally controlling for T3 recent stressful events. Because weekly nature-based physical activity minutes showed high kurtosis, additional robustness checks were conducted for the interaction term. As shown in Table 9, the interaction remained statistically significant when HC3 robust standard errors were used, when weekly minutes were winsorized at the 1st and 99th percentiles before log transformation, when observations with absolute standardized residuals greater than 3 were excluded, and when influential cases based on Cook's distance greater than 4/n were excluded. These analyses support the general pattern of results, although the continuous log-transformed physical activity index remains the preferred specification because it retains more information and reduces skewness.

**Table 8:** Sensitivity analyses.

Specification	Path	B	p	95% CI
Raw weekly minutes	NR → M	3.880	< 0.001	[2.259, 5.500]
Raw weekly minutes	PF → M	0.118	0.893	[−1.602, 1.838]
Raw weekly minutes	NR × PF → M	0.566	< 0.001	[0.235, 0.898]
Raw weekly minutes	M → Y	0.003	0.006	[0.001, 0.005]
≥150 min/week	NR → M	0.015	< 0.001	[0.008, 0.022]
≥150 min/week	PF → M	−0.000	0.925	[−0.007, 0.007]
≥150 min/week	NR × PF → M	0.001	0.036	[0.000, 0.003]
≥150 min/week	M → Y	0.627	0.023	[0.085, 1.168]
Additional T3 stress control	T2 PA (ln) → T3 PMH	0.395	< 0.001	[0.181, 0.609]
Additional T3 stress control	T3 stress event → T3 PMH	−0.030	0.744	[−0.208, 0.149]

All models controlled for sex, age, grade, growth environment, green-space accessibility, and T1 positive mental health. In the >=150 min/week specification, the mediator was coded as a binary indicator of whether weekly nature-based physical activity reached at least 150 minutes. For the additional stress-event model, T3 recent stressful event was added as an outcome-level covariate.

**Table 9:** Robustness checks for the first-stage interaction.

Specification	B	SE	*p*	95% CI	*n*
Original model 7	0.005	0.002	0.002	[0.002, 0.009]	1,191
HC3 robust SE	0.005	0.002	0.001	[0.002, 0.009]	1,191
Winsorized mediator	0.005	0.002	0.002	[0.002, 0.009]	1,191
Residual outliers excluded	0.003	0.001	0.006	[0.001, 0.005]	1,145
Cook's *d* outliers excluded	0.004	0.001	0.003	[0.001, 0.006]	1,136

Values refer to the coefficient for the T1 nature relatedness × T1 psychological flexibility interaction predicting T2 nature-based physical activity. All models controlled for sex, age, grade, growth environment, green-space accessibility, and baseline positive mental health. The winsorized mediator capped weekly minutes at the 1st and 99th percentiles before ln(minutes + 1) transformation. Residual outliers were observations with |standardized residual| > 3 in the mediator equation. Cook's D outliers were observations with Cook's distance > 4/n. HC3 = heteroskedasticity-consistent standard errors.

## 4 Discussion

This three-wave study examined whether nature-based physical activity mediated the longitudinal association between nature relatedness and later positive mental health, and whether psychological flexibility served as an exploratory first-stage boundary condition. The findings supported a baseline-adjusted longitudinal mediation pathway. T1 nature relatedness was associated with greater T2 nature-based physical activity, and T2 nature-based physical activity was associated with higher T3 positive mental health after controlling for T1 positive mental health. The indirect effect was statistically significant but small. Psychological flexibility strengthened the association between nature relatedness and nature-based physical activity, but the interaction effect was also small. Accordingly, the findings should be interpreted as evidence for a modest behavioral pathway rather than as evidence for a strong causal process.

The mediation finding extends environmental psychology research on human-nature relationships by identifying nature-based physical activity as a behavioral route linking nature relatedness with later positive mental health. Many accounts inspired by attention restoration theory and stress-recovery theory emphasize the restorative and affect-regulatory value of natural environments. The present study adds a behavioral translation perspective: students with stronger nature relatedness may be more likely to enact this orientation through outdoor or green-space physical activity, and this activity is associated with later positive mental health even after baseline positive mental health is controlled. Thus, nature relatedness may operate not only through affective appraisal or environmental preference, but also through repeated everyday behavior. This interpretation is also consistent with evidence emphasizing the importance of everyday moments of nature contact for wellbeing (Richardson et al., 2021).

The findings also clarify the theoretical meaning of positive mental health in this pathway. Positive mental health is not merely the absence of depression, anxiety, or stress. Rather, it reflects positive functioning, emotional vitality, meaning, and wellbeing. Therefore, the present findings should not be interpreted as evidence that nature-based physical activity reduces psychological symptoms. Instead, they suggest that combining physical activity with outdoor or green-space exposure may support later positive psychological functioning among university students.

Psychological flexibility should be interpreted as an exploratory boundary condition rather than a central explanatory mechanism. Psychological flexibility was not meaningfully correlated with nature-based physical activity at the bivariate level, yet it strengthened the association between nature relatedness and subsequent nature-based physical activity in the regression model. This pattern suggests that psychological flexibility may not independently make students more active in nature. Instead, it may slightly facilitate the translation of a personally meaningful connection with nature into action when students face fatigue, academic pressure, weather barriers, or low motivation. The robustness checks reduced concern that the interaction was driven solely by extreme values, but the effect size remained small.

The mediator equation explained less than 5% of the variance in nature-based physical activity, which places an important limit on the strength of the behavioral pathway. Nature relatedness is likely only one of many factors shaping students' engagement in nature-based physical activity. Structural and contextual factors, including campus green-space availability, safety, academic schedules, peer norms, weather conditions, and access to usable outdoor areas, may also contribute. This modest explanatory power reinforces the need to avoid deterministic interpretations and to view the observed pathway as one component of a broader person-environment process.

The findings have implications for campus mental health promotion from a person-environment perspective. Nature-based physical activity may integrate movement with everyday access to campus or community green spaces. However, improving green-space access or encouraging outdoor exercise may not be sufficient on its own. Universities may also need to support values-based action and flexible self-regulation so that environmental preferences and health intentions are more likely to become repeated behavior. These implications should remain tentative because the study was observational and the interaction effect was small.

Several limitations should be acknowledged. First, although the three-wave design and baseline adjustment strengthened temporal ordering, the study remains observational and cannot establish causality. Second, all focal variables were self-reported; future studies should include accelerometers, ecological momentary assessment, GPS-based exposure indicators, or behavioral logs. Third, nature-based physical activity was assessed with a brief two-item self-report index and did not distinguish specific activity types, exact intensity levels, environmental quality, biodiversity, or perceived naturalness. Therefore, outdoor or green-space location alone should be understood as a pragmatic operational criterion rather than a complete construct-validity claim. Fourth, Harman's single-factor test is a limited diagnostic for common method bias, and shared method variance cannot be fully ruled out.

Additional limitations concern longitudinal modeling and seasonality. The PROCESS-based regression approach allowed temporal ordering among the predictor, mediator, and outcome, but it does not fully exploit the complexity of longitudinal data. More advanced approaches, such as longitudinal structural equation modeling, cross-lagged panel models, or random-intercept cross-lagged panel models, could better separate within-person change from between-person differences when repeated measures of all constructs are available. The three survey waves occurred during the spring semester of 2026. Seasonal changes may have influenced outdoor activity opportunities, weather conditions, campus greenness, and exposure to natural environments. Finally, the sample consisted of Chinese university students from a single institution, which limits generalizability to other age groups, universities, regions, or cultural settings. Future studies should use multi-site samples, repeated outcome measures across all waves, and stronger longitudinal or intervention designs to examine within-person change.

## 5 Conclusion

This baseline-adjusted three-wave longitudinal study found that T1 nature relatedness was longitudinally associated with T3 positive mental health partly through T2 nature-based physical activity among Chinese university students. Psychological flexibility showed a small first-stage moderating effect, suggesting that it may function as an exploratory boundary condition in the translation of nature relatedness into nature-based physical activity. Overall, the findings point to a modest behavioral pathway linking human-nature connection, nature-based physical activity, and later positive mental health. Because the effects were small, the mediator model explained limited variance, and the study was observational, the conclusions should not be interpreted as causal evidence.

## Data Availability

The data supporting the conclusions of this article will be made available by the corresponding author, upon reasonable request.
